# Supplementary material for: Electrochemically-stable ligands bridge the photoluminescence-electroluminescence gap of quantum dots
Source: Nat Commun. 2020 Feb 18;11:937. doi: 10.1038/s41467-020-14756-5 (PMC7028909; doi:10.1038/s41467-020-14756-5)
Supplement: Supplementary file 1 — Supplementary Information [file 41467_2020_14756_MOESM1_ESM.pdf]

Supplementary Information for

Electrochemically-stable ligands bridge the photoluminescence-electroluminescence gap of quantum dots

Pu *et al.*

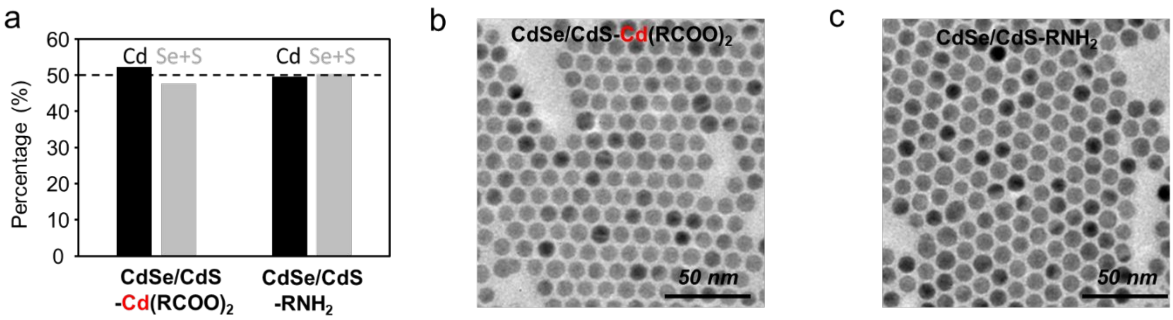

Supplementary Figure 1. Chemical and structural characterizations of the QDs. a, Atomic ratios of Cd and (Se + S) of the QDs, confirming that the ratio of cadmium to anions (Se+S) for the CdSe/CdS-RNH_2 QDs is stoichiometric within experimental error and the CdSe/CdS-$Cd(RCOO)_2$ QDs possess excess cadmium ions. **b** and **c,** Transmission electron microscope images of the QDs. Scale bar: 50 nm.

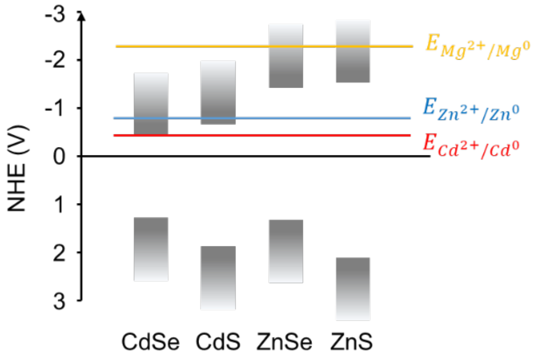

Supplementary Figure 2. Energy level diagram of several bulk semiconductors. Standard reduction potentials of Cd^{2+}/Cd^0, Zn^{2+}/Zn^0 and Mg^{2+}/Mg^0 are also shown.

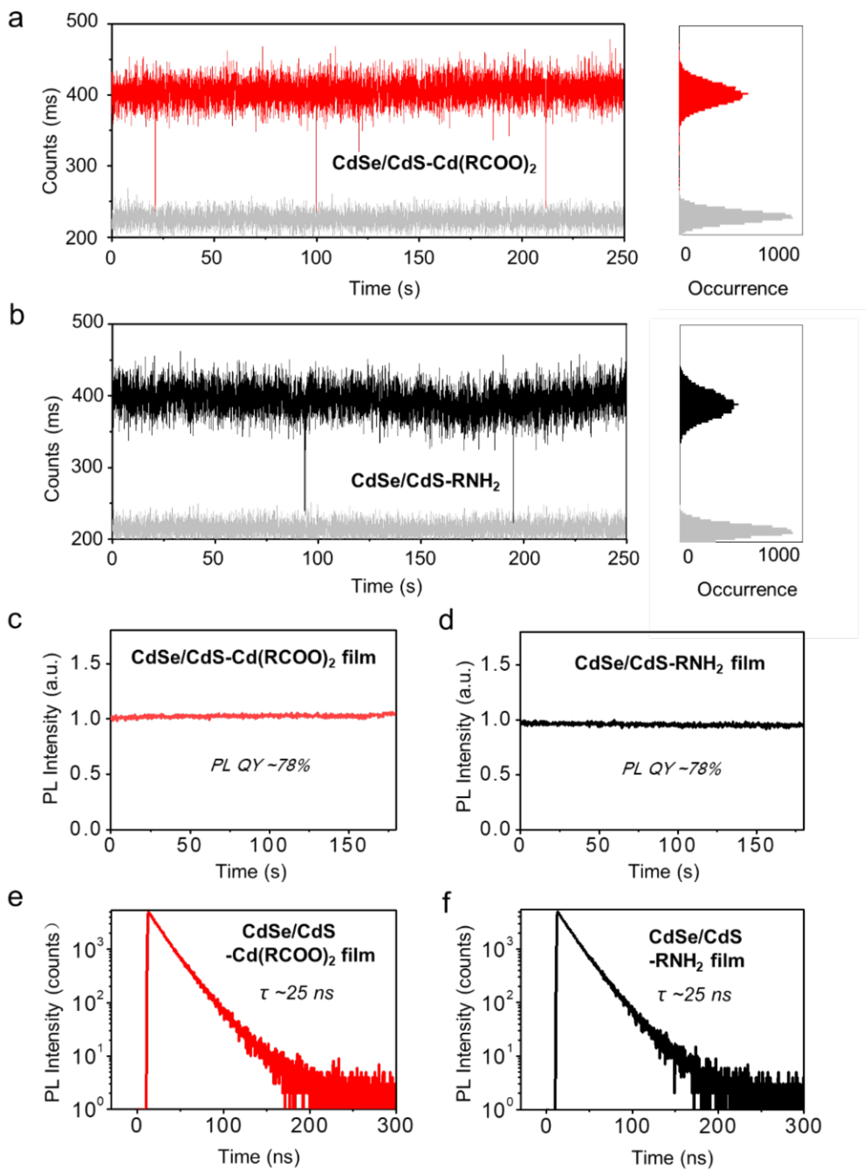

Supplementary Figure 3. Photoluminescence properties of the QDs. a and **b,** Photoluminescence intensity-time traces of single CdSe/CdS-Cd(RCOO)$_2$ and CdSe/CdS-RNH$_2$ QD, respectively, indicating non-blinking characteristics. **c** and **d,** Stable and efficient photoluminescence of two types of QDs in thin films. **e** and **f,** The corresponding time-resolved photoluminescence decay curves for both types of QDs in thin films.

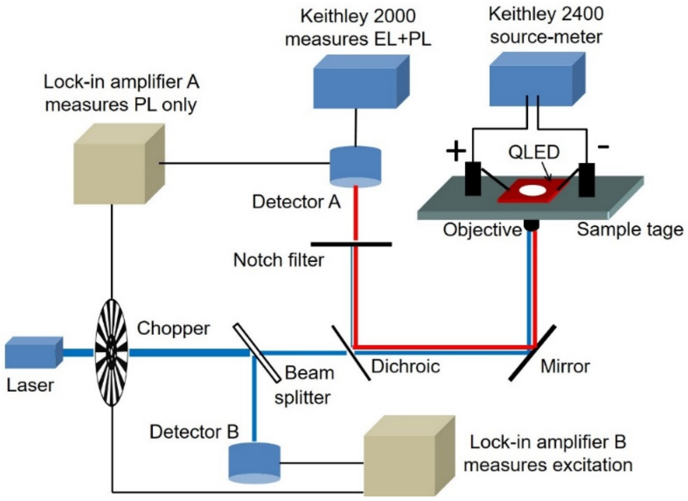

Supplementary Figure 4. Experimental Setup for the simultaneous PL-EL measurements. The equipment operando monitors the relative changes of photoluminescence efficiency of QDs in working devices by using low-frequency chopped, low-intensity photo-excitation**.**

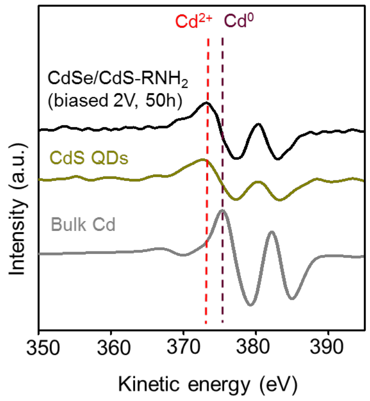

Supplementary Figure 5. Differential Auger electron spectra. The signal of CdSe/CdS-RNH_2 QDs (biased in QLEDs) and two control samples of CdS nanocrystals and metal Cd foils are shown.

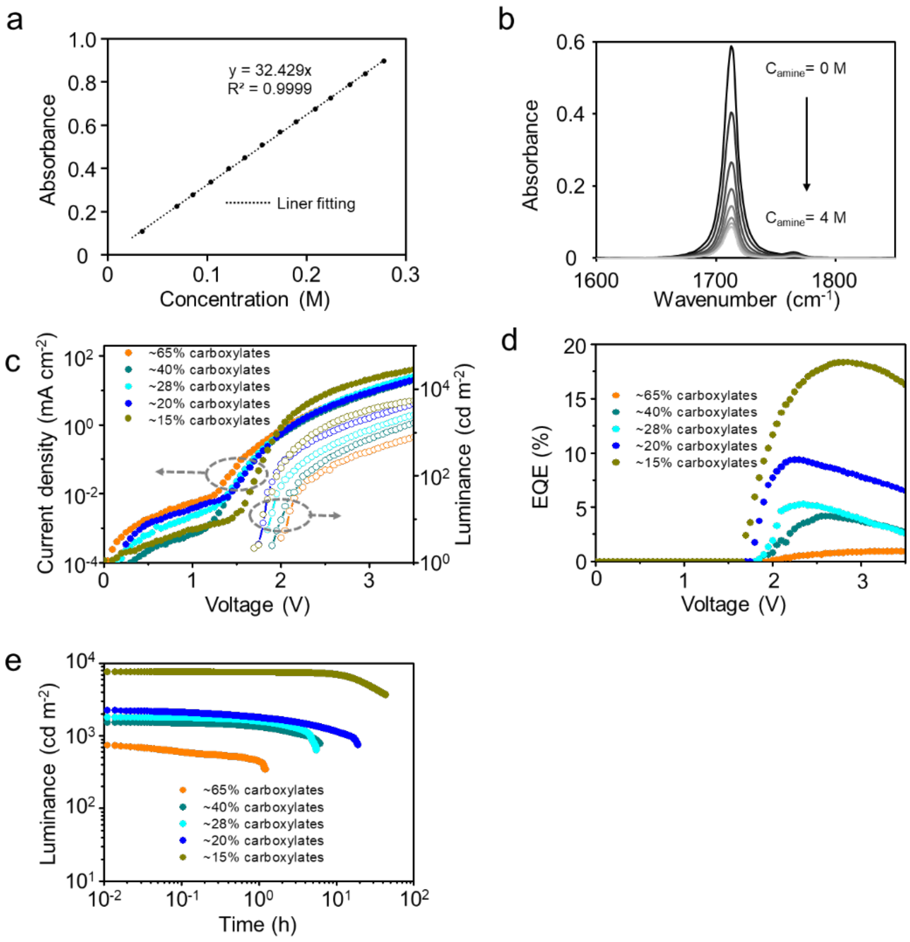

Supplementary Figure 6. Ligand replacement and concentration-dependent EL properties. a, FTIR calibration curve for measuring the concentration of fatty acids by using the absorbance of the C=O vibration of fatty acids. **b,** FTIR spectra of the fatty acids obtained by digesting the QDs with hydrochloric acid (see experimental for details). **c, d,** and **e,** J-L-V, EQE-V and stability data of the QLEDs based on the QDs with different percentages of residual carboxylates.

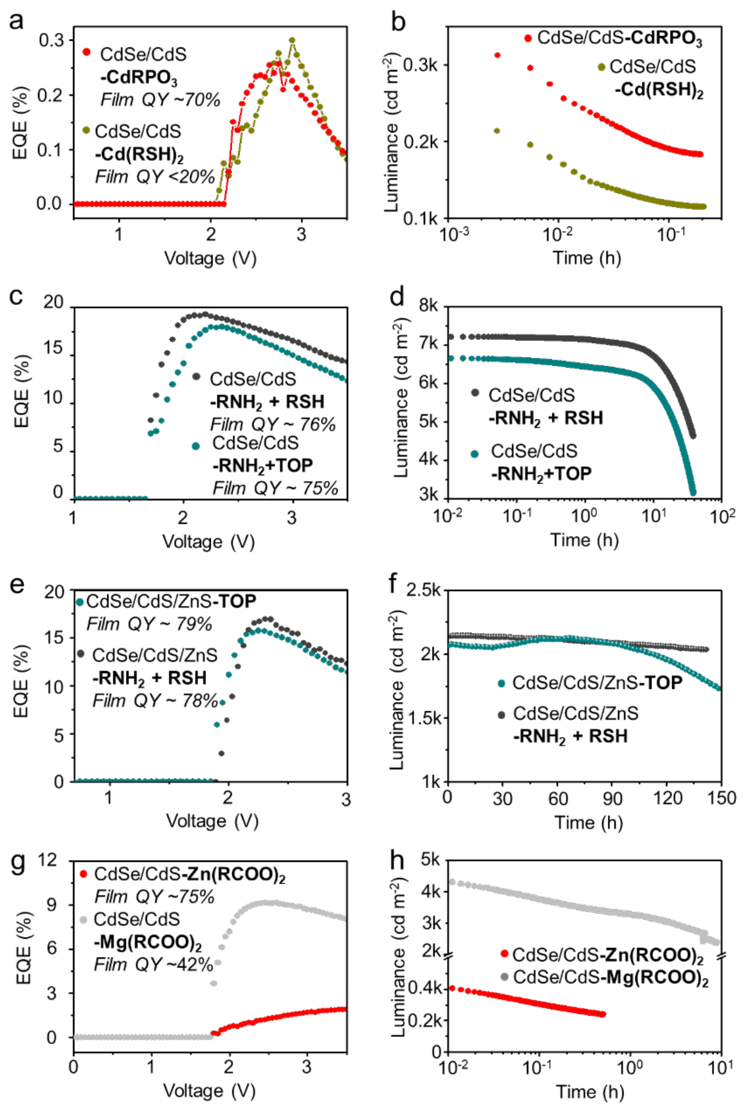

Supplementary Figure 7. Characteristics of QLEDs based on the QDs with different ligands. Photoluminescence QY of thin films is provided for each sample in the plot. **a** and **b**, CdSe/CdS core/shell QDs with cadmium phosphonate ligands (Cd(RPOOO)) or cadmium thiolate ligands ($Cd(RS)_2$). **c** and **d**, CdSe/CdS core/shell QDs with mixed ligands of amine and TOP (RNH_2 + TOP) or amine and thiol (RNH_2 + RSH). **e** and **f**, CdSe/CdS/ZnS core/shell/shell QDs with TOP ligands or mixed ligands of amine and thiol (RNH_2 + RSH). **g** and **h**, CdSe/CdS core/shell QDs with zinc-carboxylate ($Zn(RCOO)_2$) ligands or magnesium-carboxylate ($Mg(RCOO)_2$) ligands.

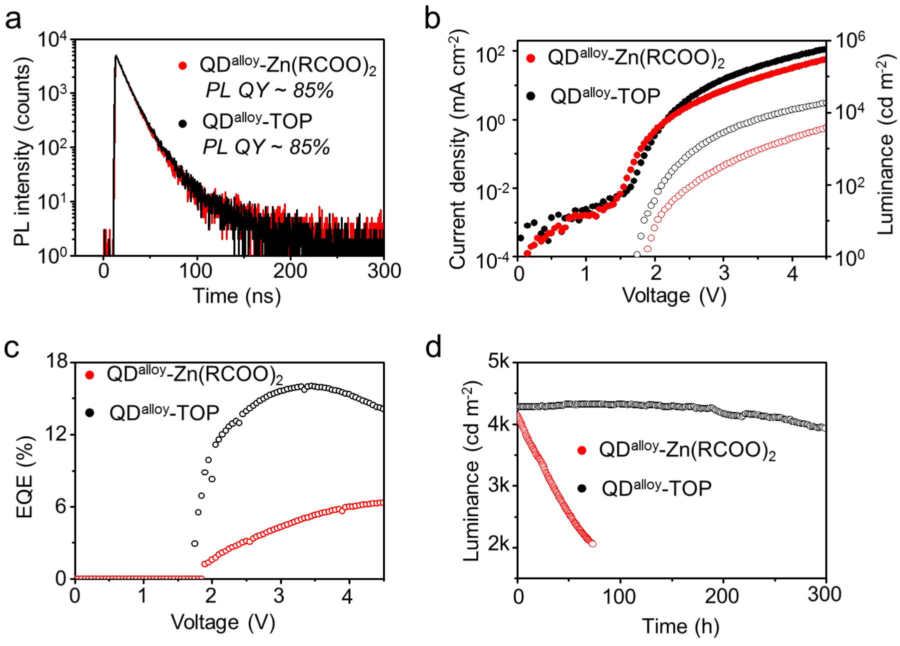

Supplementary Figure 8. PL and EL properties of the CdSe/CdZnSe/CdZnS QDs. a, Transient photoluminescence spectra. **b-d,** J-L-V, EQE-V and stability data of the QLEDs based on the two types of QDs.

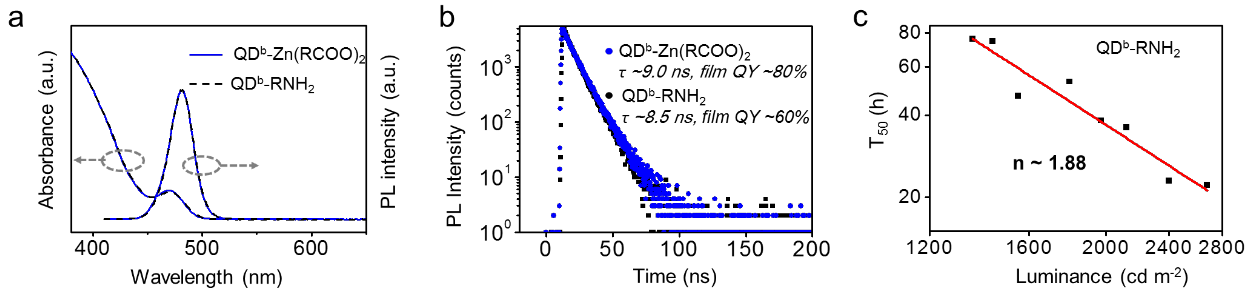

Supplementary Figure 9. PL and EL properties of the blue CdSeS/ZnSeS/ZnS QDs. a, Absorption and steady-state photoluminescence spectra. **b,** Transient photoluminescence spectra of the QDs in solution. The photoluminescence QY of QDs in film are also shown. **c,** Operational lifetime of the blue QLEDs using the CdSeS/ZnSeS/ZnS-RNH_2 QDs measured at different initial brightness. These devices were tested under a constant-current mode. The data are fitted by an empirical equation, $L_0^n \times T_{50}$ = constant, leading to an acceleration factor of 1.88.

Supplementary Table 1 Comparison of the state-of-the-art QLEDs in literature

QD structure	Ligands in the synthetic system	EL peak (nm)	Device structure	Turn-on voltage (V) @1 cd m^{-2}	Peak EQE (%)	Lifetime (h) T_{50}@ 100 cd m^{-2}	Ref.
CdSe/CdS	**Decylamine & Dodecanethiol**	640	ITO/PEDOT:PSS/P-TPD/ PVK/QDs/PMMA/ZnO/Ag	1.7	20.5	100,000	1
CdSe/CdZnS	Oleic acid & **TOP**	624	ITO/PEDOT:PSS/P-TPD/ PVK/QDs/ZnMgO/Ag	1.7	18.2	190,000	2
CdSe/CdS	Oleic acid & **TOP & 1-dodecanethiol**	658	ITO/ZnO/QDs/CPB/ MoO_x/Al	1.9	11.36	______	3
CdSe/$Cd_{1-x}Zn_xSe_{1-y}S_y$/ ZnS	Oleic acid & **TOP**	631	ITO/PEDOT/TFB/QD/ZnO/Al	1.7	15.1	2,200,000	4
CdSe/$Cd_{1-x}Zn_xSe$/$ZnSe_yS_{1-y}$	Oleic acid & **TOP**	611	ITO/ZnO-PVP/QDs/TCTA/ MoO_x/Al	1.9	13.5	1,330,000	5
CdSe@ZnSe/ ZnS	Oleic acid & **TOPO**	537	ITO/PEDOT:PSS /TFB/ QDs/ZnO/Al	2.3	14.5	90,000	6
CdSe@ZnS/ ZnS	Oleic acid & **TOP**	~520	ITO/ZnO/QDs/PEIE/P-TPD/ MoO_x/Al	~3.2	15.6	______	7
$Zn_xCd_{1-x}S_ySe_{1-y}$	Oleic acid & **TOP**	526	ITO/PEDOT:PSS/TFB/ QDs/ZnO/Al	2.2	21	T_{90} @ 2500 nits 490	8
CdZnSeS@ ZnS	**TOP & 1-dodecanethiol**	~540	ITO/PEDOT/TFB/QDs/ ZnMgO/Al	2.0	16.6	——	9
ZnCdSe/ZnS// ZnS	**Trioctylamine & TOP**	479	ITO/PEDOT/TFB/QDs/ PMMA/ZnO/Al	2.4	16.2	355	10
$Cd_{1-x}Zn_xS$/ ZnS	Oleic acid & **TBP**	467	ITO/PEDOT/TFB/QDs/ ZnO/Al	~2.4	~7	T_{50} @ 1000 nits 23	11

References

1. Dai, X. L. *et al.* Solution-processed, high-performance light-emitting diodes based on quantum dots. *Nature* **515**, 96-99, (2014).
2. Zhang, Z. X. *et al.* High-Performance, Solution-Processed, and Insulating-Layer-Free Light-Emitting Diodes Based on Colloidal Quantum Dots. *Adv. Mater.* **30**, 1801387, (2018).
3. Cho, I. *et al.* Multifunctional Dendrimer Ligands for High-Efficiency, Solution-Processed Quantum Dot Light-Emitting Diodes. *Acs Nano* **11**, 684-692, (2017).
4. Cao, W. R. *et al.* Highly stable QLEDs with improved hole injection via quantum dot structure tailoring. *Nat. Commun.* **9**, 2608, (2018).
5. Lim, J., Park, Y. S., Wu, K. F., Yun, H. J. & Klimov, V. I. Droop-Free Colloidal Quantum Dot Light-Emitting Diodes. *Nano Lett.* **18**, 6645-6653, (2018).
6. Yang, Y. X. *et al.* High-efficiency light-emitting devices based on quantum dots with tailored nanostructures. *Nat. Photonics* **9**, 259-266, (2015).
7. Kim, D. *et al.* Polyethylenimine Ethoxylated-Mediated All-Solution-Processed High-Performance Flexible Inverted Quantum Dot-Light-Emitting Device. *ACS Nano* **11**, 1982-1990, (2017).
8. Acharya, K. P. *et al.* High efficiency quantum dot light emitting diodes from positive aging. *Nanoscale* **9**, 14451-14457, (2017).
9. Sun, Y. *et al.* Investigation on Thermally Induced Efficiency Roll-Off: Toward Efficient and Ultrabright Quantum-Dot Light-Emitting Diodes. *ACS Nano* **13**, 11433-11442, (2019).
10. Lin, Q. *et al.* Nonblinking Quantum-Dot-Based Blue Light-Emitting Diodes with High Efficiency and a Balanced Charge-Injection Process. *ACS Photonics* **5**, 939-946, (2018).
11. Chen, S. *et al.* On the degradation mechanisms of quantum-dot light-emitting diodes. *Nat Commun* **10**, 765, (2019).
